# Molecular studies on ancient *M. tuberculosis* and *M. leprae*: methods of pathogen and host DNA analysis

**DOI:** 10.1007/s10096-015-2427-5

**Published:** 2015-07-26

**Authors:** H. W. Witas, H. D. Donoghue, D. Kubiak, M. Lewandowska, J. J. Gładykowska-Rzeczycka

**Affiliations:** Department of Molecular Biology, Medical University of Łódź, Łódź, Poland; Division of Infection and Immunity, University College London, London, UK; Retired professor, Marusarzówny, Gdańsk, Poland

## Abstract

Humans have evolved alongside infectious diseases for millennia. Despite the efforts to reduce their incidence, infectious diseases still pose a tremendous threat to the world population. Fast development of molecular techniques and increasing risk of new epidemics have resulted in several studies that look to the past in order to investigate the origin and evolution of infectious diseases. Tuberculosis and leprosy have become frequent targets of such studies, owing to the persistence of their molecular biomarkers in ancient material and the characteristic skeletal lesions each disease may cause. This review examines the molecular methods used to screen for the presence of *M. tuberculosis* and *M. leprae* ancient DNA (aDNA) and their differentiation in ancient human remains. Examples of recent studies, mainly from Europe, that employ the newest techniques of molecular analysis are also described. Moreover, we present a specific approach based on assessing the likely immunological profile of historic populations, in order to further elucidate the influence of *M. tuberculosis* and *M. leprae* on historical human populations.

## Introduction

**Fig. 1:**
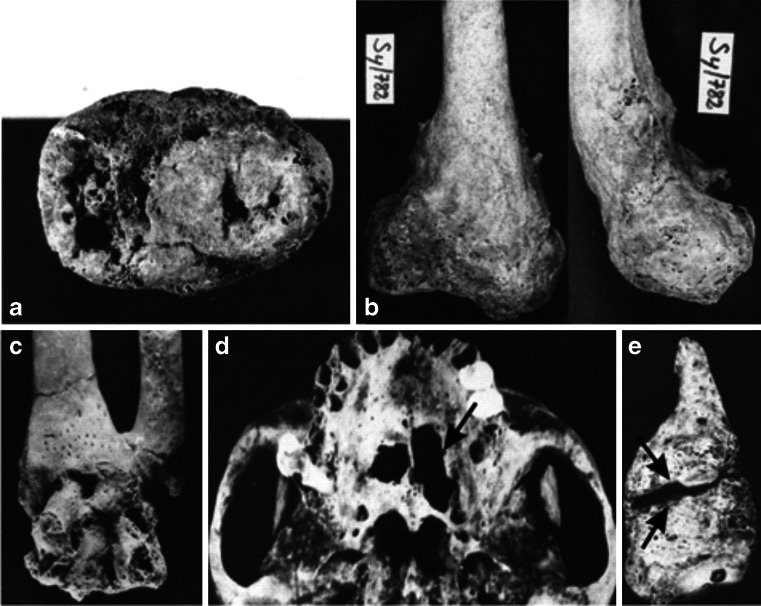
Lesions typical of tuberculosis: **a** defects of proximal epiphysis of the tibia seen from above, **b** distorted distal end of the femur (Sypniewo, northeast Poland), **c** changes of the distal end of the left forearm and carpus (Ołbin in Wrocław, southwest Poland); lesions typical of leprosy: **d** defects in the palate, **e** fusion of the Lisfranc joint and atrophy of the first metatarsal bone (Suraż, northeast Poland) [4]

Infectious diseases such as tuberculosis, plague, leprosy and cholera have accompanied humans since the dawn of our history. The past is marked by epidemics and pandemics that have influenced all aspects of human life. Scientific discoveries and significant development of techniques of molecular biology in recent years have caused increasing interest in investigating the origins and history of infectious diseases and their agents. Studying ancient diseases is essential for understanding microbial evolution, spread, and confirmation of epidemics and pandemics. Tuberculosis and leprosy are among the diseases that arouse the greatest interest in paleopathologists, as they may leave characteristic lesions on the bones that suggest a diagnosis based on bone morphology. In osteoarticular tuberculosis, skeletal lesions are most often found in the thoracic and lumbar vertebrae, as well as hip and knee joints. Tuberculosis can also attack the epiphysis and metaphysis of the long bones (Fig. 1a–c). Lesions on the internal surface of ribs are another indicator of possible skeletal tuberculosis but do not provide firm identification [1, 2]. Changes considered as indicative of leprosy include the rhinomaxillary syndrome (*Facies leprosa*) (Fig. 1d) and lesions of the hand, foot, tibia and fibula (Fig. 1e). New bone formation in the peritoneum of tibia and fibula alone are not accepted as criteria for leprosy diagnosis due to the fact that a number of conditions can cause those changes [2, 3].

Both tuberculosis and leprosy are caused by mycobacteria, which have characteristic, lipid-rich thick cell walls that are less susceptible to degradation [5] and can be detected directly, without amplification [6, 7]. These lipid biomarkers are totally distinct from anything found in mammalian tissue and are specific for members of the *Mycobacterium tuberculosis* complex (MTBC) or *M. leprae*, so enable independent identification of these pathogens in ancient material at the molecular level. Molecular markers not only allow the verification of paleopathological diagnoses but also enable individuals with ambiguous or no visible lesions to be screened for the presence of bacterial biomolecular markers, since, in the majority of tuberculosis and leprosy cases, there are no visible bone changes [8, 9].

Tuberculosis and leprosy are fatal diseases that still cause deaths in both developed and developing countries. It is a curable and preventable disease that most often affects lungs, caused by members of a group of closely related bacterial species, which comprise the MTBC. It is the greatest killer worldwide due to a single infectious agent. Data obtained from 202 countries and territories show that, in 2013, 9.0 million people were infected with *M. tuberculosis*, 1.5 million died because of tuberculosis, while about one-third of the world’s population has latent tuberculosis [10]. Leprosy is a curable, chronic disease caused by *Mycobacterium leprae*. This disease mainly affects skin, peripheral nerves, mucosa of the upper respiratory tract and eyes. According to official data from 102 countries, 215,656 new cases of leprosy were reported in 2013 [11] and it is still the greatest cause of infection-related disability in the world.

The earliest known paleopathological evidence of human tuberculosis was found in ancient Syria at around the time (8800–7600 BC) of animal domestication [12]. Before this study, the earliest evidence was from two Neolithic individuals (7000 BC) in the Eastern Mediterranean [13]. Other ancient cases of tuberculosis described in the literature include a 5400–4800 BC case from central Germany [14], a 5000 BC case from Hungary [6] and a 4000 BC case from Italy [15, 16]. The first molecular studies of ancient infectious diseases used the method of collecting samples and testing for the presence of MTBC ancient DNA (aDNA) described by Spigelman and Lemma [17]. In 1994, the first report of tuberculosis based on tissue from a pre-Columbian Peruvian mummy was published, including confirmatory aDNA cloning and sequencing [18]. The earliest known paleopathological evidence of leprosy came from India from the post-urban phase of the Indus Age [19]. A number of other ancient cases of leprosy have been described in the literature, including a 4th–3rd century BC case from Bologna, northeastern Italy [20], a 4th century BC–3rd century AD case from Thailand [21] and a 3rd century BC case from Britain [22]. The first molecular evidence of *M. leprae* was published in 1994 [23, 24] and the oldest sample confirmed by polymerase chain reaction (PCR) was from the 1st century AD [25]. The present article is a review of methods used for the identification and differentiation of the MTBC and *M. leprae* DNA in ancient human remains. Furthermore, we present a number of tuberculosis and leprosy cases, mainly from Europe, in samples recently excavated from archaeological sites and those housed in museum collections that have been subjected to molecular analysis. Finally, we propose further approaches for assessing the possible presence of bacteria and their influence on ancient humans, mainly by estimation of the immunological profiles of historic populations.

## Methods of molecular identification and differentiation of *M. tuberculosis* and *M. leprae*

### Properties of aDNA

Spontaneous hydrolysis and oxidation are examples of the most commonly found damage that cause post mortem instability of nucleic acids. DNA decay typically demonstrates baseless sites, double- and single-strand breaks, miscoding lesions and cross-links [26–29], although the relative rates of different kinds of aDNA damage and their mode of accumulation vary between specimens under different environmental conditions [30]. Since it is almost impossible to retrieve long amplification products of aDNA, researchers focus on short sequences, around 100–200 base pairs (bp), and repetitive regions. Real-time PCR, based on the incorporation of fluorescent markers into double-stranded DNA, is especially useful in this kind of research because of the ability to target shorter regions that can be visualised on screen instead of on a gel. Genomic deletions can be examined by PCR followed by gel electrophoresis. Detection of single nucleotide polymorphisms (SNPs) by PCR-restriction fragment length polymorphism (PCR-RFLP) analysis is based on sequencing of the PCR product or its digestion with a restriction enzyme and subsequent gel electrophoresis. The problems arising from degradation and contamination of the samples are much reduced in methodology such as next-generation sequencing (NGS) [31] or whole-genome sequencing (WGS) [32]. In NGS, selective enrichment of target DNA can be achieved by the use of microarrays or bead suspensions that are able to bind specific target sequences. An alternative approach in WGS is ‘shotgun’ sequencing, where every DNA fragment is tagged and sequenced. Thereafter, high-throughput sequencing and bioinformatic analysis is performed [33, 34].

### Molecular identification and differentiation of the MTBC and *M. tuberculosis*

**Fig. 2:**
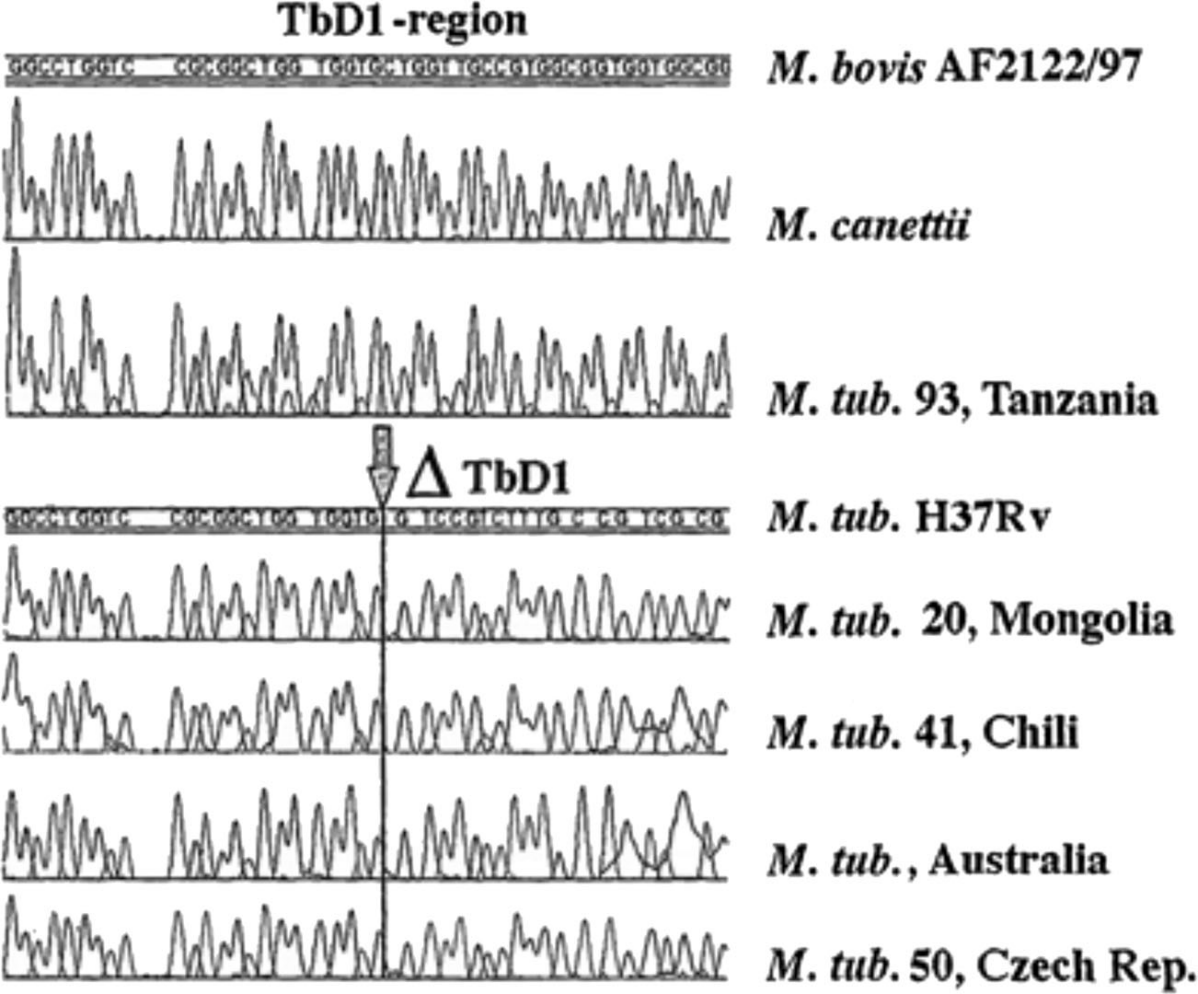
Sequences in the TBD1 region received from strains of different geographic regions [45]

The MTBC consists of a group of very closely related species, including *M. tuberculosis*, *M. africanum*, *M. bovis*, *M. microti*, *M. pinnipedii* and *M. canettii* [35, 36] that cause infections in humans and animals. Identification of the MTBC at the species level, as well as differentiation of *M. tuberculosis* genotypes derived from skeletal samples, may be performed using the molecular methods described below. In the MTBC, there are specific regions of the repetitive element IS*6110* that can identify these pathogenic lineages. In human *M. tuberculosis*, the number of copies of IS*6110* varies between strains, from 0 in rare strains from Southeast Asia to 24 copies/cell, while in other members of the MTBC, there is only one copy of this element per cell [37]. The specific IS*6110* primers have been used frequently as a marker of the presence of MTBC DNA but it has long been known that different regions of this repetitive element have been detected in mycobacteria other than tuberculosis [38]. The repetitive element IS*1081* is also used to detect DNA from the MTBC and, as there are normally six copies per cell, it has been used for quantitative analysis and for the detection of *M. bovis* [39]. Other examples used for the detection of the MTBC include specific sites in the RNA polymerase beta gene (*rpoB*) and 19 kDa antigen gene [40–42]. Evolutionary ‘modern’ and ‘ancient’ strains of *M. tuberculosis* can be distinguished by analysis of the *M. tuberculosis*-specific D1 deletion site (Fig. 2). Loss of the TbD1 region is common in strains from all three major groups of *M. tuberculosis* described by Sreevatsan et al. [43], in contrast to all strains of *M. bovis* and in a part of the early group 1 lineage of *M. tuberculosis* [44].

Since the entire genome of *M. tuberculosis* and *M. bovis* have been sequenced, there is a wide choice of genetic sites that can be used to distinguish between these species. For example, *M. tuberculosis* and *M. bovis* can be distinguished from *M. bovis* based on the deletion region RD2 (now known as RD7) that is absent in *M. bovis* [45, 46]. Spoligotyping (spacer oligonucleotide typing) is based on the variation of the direct-repeat (DR) locus found in the members of the MTBC that enables the differentiation of the members of the MTBC and identification of *M. tuberculosis* strains. This region contains multiple, well-conserved 36 bp (DRs) interspersed with non-repetitive spacers of 34 to 41 bp. Members of the MTBC and strains of *M. tuberculosis* vary in the number of DRs and in the absence or presence of particular spacers [47]. The spacer regions can be lost over time, so this method is suitable for investigating evolutionary aspects of human tuberculosis and has also been used to clarify the origin and transmission of this disease in different time periods and populations. The MTBC can be assigned to four genotypic groups, known as principal genetic groups (PCGs) (Table 1), based on the combination of polymorphisms at codons 463 and 203 of the *katG* gene and codon 95 of the *gyrA* gene [43, 48].

**Table 1:** Genetic groups of the MTBC based on the mutations in the *katG* and *gyrA* codons [43, 44, 48]

	Genetic group			
Gene codon	1A	1B	2	3
*katG* 463	CTG (Leu)	CTG (Leu)	CGG (Arg)	CGG (Arg)
*katG* 203	ACT (Thr)	ACC (Thr)	ACC (Thr)	ACC (Thr)
*gyrA* 95	ACC (Thr)	ACC (Thr)	ACC (Thr)	AGC (Ser)
Member of MTBC	MTBC precursor *M. africanum* group A, *M. bovis*, *M. microti*	Ancestral *M. tuberculosis* *M. africanum* group B	*M. tuberculosis*	*M. tuberculosis*

Differentiation of the species of the MTBC can now be performed by typing based on synonymous SNPs that are functionally neutral, so can be used to distinguish between lineages, aided by the lack of horizontal gene transfer. Initially, SNP-based phylogenetic analysis of a global collection of *M. tuberculosis* isolates classified the MTBC into six phylogenetically distinct groups, called lineages or SNP cluster groups (SCGs), with a seventh group containing all *M. bovis* isolates [49]. Subsequently, this led to the recognition of seven phylogeographic lineages, each associated with specific human populations, with the animal lineages sometimes described as lineage 8 [50]. The results of WGS [50] as well as genotyping and selected sequencing, for example, large sequence polymorphisms (LSP) [51], suggest that each lineage is linked with a restricted geographical distribution. Lineage 1 occurs predominantly in the Philippines and Indian Ocean, lineage 2 in East Asia and lineage 3 in India as well as East Africa. In Europe and both Americas, lineage 4 is most likely to be observed, while lineage 5 occurs mostly in West Africa [51, 52]. Lineage 7 has, so far, only been observed in Ethiopia and recent Ethiopian emigrants [53].

### Methods of molecular identification and differentiation of *M. leprae*

All extant isolates of *M. leprae* are nearly indistinguishable, being derived from a single clone. The *M. leprae* genome has been shaped by reductive evolution and mass pseudogene formation that has moulded its 3.31-Mb genome. Molecular genotyping and sub-genotyping of *M. leprae* can be performed using methods based on SNPs. All isolates of *M. leprae* can be assigned to one of four SNP types determined by four combinations of three SNPs and 16 SNP subtypes (Table 2) based on SNPs and InDel repeat sequences [54, 55]. Due to being an uncultivable obligate pathogen, isolates of *M. leprae* exhibit a strong relationship between their SNP subtypes and geographical locations that reflects patterns of early human migrations and trade routes, including the Silk Road that linked Europe and China. Thereby, SNP typing provides evidence for disease origins, dissemination and phylogeny [55].

**Table 2:** *M. leprae* SNP types by Monot et al. [54, 55]

	SNP type			
	1	2	3	4
Position in genome				
14676	C	C	C	T
1642875	G	T	T	T
2935685	A	A	C	C
Subtypes	A, B, C, D	E, F, G, H	I, J, K, L, M	N, O, P
Present occurrence	Asia, the Pacific region, East Africa	Ethiopia, Malawi, Nepal/North India, New Caledonia	North Africa, Americas	West Africa, the Caribbean region

Variable number tandem repeats (VNTR) typing is a method based on amplifying and sequencing of polymorphic microsatellite and minisatellite loci (short tandem repeat motif, STR) present in the *M. leprae* genome. Its usefulness in modern cases is well proven. The *rpoT* locus (ML1022c) was the first investigated VNTR locus in the *M. leprae* genome non-coding region. Two variants of a 6-bp tandem repeat, composed of GACATC, have been reported, one of four and one of three tandem repeats [56]. TTC (AGA)20 locus (ML2344–ML2345) polymorphisms were also identified from the *M. leprae* genome sequence database. The number of repeats at each locus may vary among *M. leprae* strains, while most of the sequences in *M. leprae* seem to be conserved. Sequence analysis of the TTC repeat region in each of the *M. leprae* strains shows a variation of 10 to 37 repeats in the non-coding region. The most reliable explanation of the evolution of *M. leprae* strains with different numbers of TTC repeats is slippage or addition of one codon during replication [57, 58]. Latterly, over 40 potential variable loci (33 microsatellites and 11 minisatellites) have been identified. Multiple loci variable number tandem repeats analysis (MLVA) can be used for investigating cases within a limited geographic area, to distinguish strains with the same SNP profiles.

### Use of molecular identification and differentiation in cases of ancient tuberculosis

In the first paper published on the detection of ancient tuberculosis by molecular analysis, IS*6110* was the target used [17, 18]. In a significant early study in 2002, six cases dated to 7th–8th century AD and eight cases dated to the 17th century AD, from the collections housed at the Department of Anthropology at the University of Szeged, Hungary, were screened [59]. All examined individuals exhibited signs of infection on the bones. The presence of mycobacterial DNA was investigated by amplifying the gene encoding the 65 kDa antigen, although this target is of doubtful use as it is found in all environmental mycobacteria. However, the presence of MTBC DNA was demonstrated by the amplification of a segment of the repetitive sequence IS*6110*. In several cases, the PCR product of IS*6110* was treated with *Hae*III to demonstrate MTBC typical digestion products. Direct sequencing was applied to establish the nucleotide sequences. The presence of mycobacterial DNA was confirmed in 13 cases, while IS*6110* PCR was positive in eight cases [59].

In England, nine medieval human burials with signs of tuberculosis, from Wharram Percy, were screened [60]. PCR analysis of IS*6110* and *rpoB* enabled verification of the paleopathological diagnosis of tuberculosis. Conventional PCRs for *mtp-40*, *oxyR*, *pncA* and the RD7 deletion were performed to distinguish between *M. tuberculosis* and *M. bovis*. Since the skeletal lesions of brucellosis may resemble those of tuberculosis, PCR analysis for *Brucella* DNA was also undertaken with the use of the IS*6501* multi-copy insertion element, variable among species of *Brucella* (5–35 repeats in the genome) but was negative. The results (Table 3) indicate that, in all nine tuberculosis cases from Wharram Percy, *M. tuberculosis* DNA rather than *M. bovis* DNA was present. Spoligotyping was also used here to detect and type members of the MTBC. This can also elucidate the origin and transmission of the pathogen in different time periods and populations [61].

**Table 3:** Genotyping of nine medieval individuals with signs of tuberculosis from Wharram Percy [60]

	IS*6110*	*rpoB*	*mtp-40*	*oxyR*	*pncA*	RD7 deletion	Spoligotyping	IS*6501* ^a^
Controls								
EE003	−	−	nd	nd	nd	nd	nd	nd
G304	−	−	nd	nd	nd	nd	nd	nd
Samples								
EE056	+	+	+	−	nd	nd	−	−
G438	+	+	+	+ (G)	nd	−	nd	−
G482	+	+	+	+ (G)	nd	−	−	−
NA026	+	+	−	+ (G)	nd	−	*Mtb*	−
NA046	+	nd	−	+ (G)	nd	−	*Mtb*	−
NA112	−	+	+	+ (G)	nd	nd	*Mtb*	−
NA197	+	+	+	−	nd	−	−	−
SA013	−	+	+	+ (G)	nd	−	−	−
WCO142	+	+	+	−	nd	−	−	−

− = no result; + = positive result; nd = not determined

^a^ *Brucella*-specific insertions (5–35 repeats in the genome)

In ancient Egypt, Zink and colleagues reported their analysis of bone and tissue samples from 85 Egyptian mummies, dated to 2050–500 BC, found in different tomb complexes of West Thebes, Upper Egypt [61]. Samples were screened for the presence of MTBC DNA, using IS*6110* PCR. Those yielding positive results for Mbov, *mtp-40* and the *oxyR* regions were amplified to differentiate between *M. tuberculosis* and *M. bovis*. Furthermore, in those samples, spoligotyping was used for the subsequent analysis of *M. tuberculosis* DNA. IS*6110* PCR confirmed the presence of MTBC DNA in 25 samples, although PCR methods to distinguish *M. tuberculosis* from *M. bovis* gave inconclusive results, as summarised in Table 4. Although five modern samples were positive with the *M. bovis*-specific primers, only two ancient samples were successfully amplified for *mtp-40*. The *oxyR* gene was detected in only three ancient samples.

**Table 4:** Results of PCR-based differentiation between *M. tuberculosis* and *M. bovis* [62]

	*M. bovis*	*mtp-40-*1/2	*mtp-40-*3/4	*oxyR*	IS*6110*	Spoligotyping
Modern control DNA						
*Mtb* H37Rv (10 ng/μl)	+	+	+	+	+	*Mtb*
*Mb* BCG (10 ng/μl)	+	+	+	+	+	*Mb*
*Mtb* H37Rv (0.1 ng/μl)	−	+	+	+	+	*Mtb*
*Mb* BCG (0.1 ng/μl)	+	−	+	+	+	*Mb*
Recent autopsy cases						
Paraffin sample *Mb*	+	−	−	−	+	*Mb*
Paraffin sample *Mtb*	−	−	−	−	+	*Mtb*
Historic tissue samples						
TT453-9	+	−	−	−	+	*Mtb*
TT453-14	+	−	−	−	+	*Mtb*
TT95-122	+	−	−	+	+	*Mtb*
TT95-40	−	−	−	+	+	*Mtb*
TT95-169	−	−	−	−	+	*Mtb*
DAN93-11	+	+	−	−	+	*Mtb*
DAN95.1-1	+	−	+	+	+	*Mtb*
TT196-M5	−	−	−	−	+	*Mafr*

− = no result; + = positive result; Mtb = *M. tuberculosis*; Mb = *M. bovis*; Mafr = *M. africanum*

During spoligotyping, due to the degradation of the aDNA, all blots were performed in triplicate. Reproducible patterns were obtained from 12 individuals and compared to those in the international spoligotyping database. Three isolates matched types 53, 393 and 291 from the database and are close or identical to the ubiquitous *M. tuberculosis* type 53. Type 53 represents one of the most common profiles. Five samples showed a pattern similar to that of *M. africanum*. Others were not found in the database, but all of them were related to the *M. tuberculosis* TbD1-deleted type, suggested by the lack of spacers 33 to 36 and the presence of spacers between position 39 to 45. Patterns suggestive for *M. bovis* were not found in any of the examined samples. This study is an early example of paleoepidemiology and was made possible by the excellent preservation of the skeletal material in Ancient Egypt [62, 63].

A remarkable collection of 263 naturally mummified bodies was discovered in an 18th century AD Hungarian crypt in Vác, Hungary in 1994–1995. In many cases, there were archival records giving details of name, family connections, date of death and, occasionally, information on occupation or symptoms. Because of the local environmental conditions, the human remains were in an excellent state of preservation. In an initial study [64], 350 samples from 168 individuals were examined. A two-tube nested PCR of the IS*6110* insertion sequence was used in the initial screening. Polymorphisms at codon 463 of *katG* and codon 95 of *gyrA* were assessed in order to place the strains into genotypic groups [43]. Methods specific for *M. tuberculosis* were positive in 55 % of the samples. Initial PCR-based genotyping showed that two samples were classified as PGG2, five samples were classified as PGG3, while five other samples were classified as not PGG1 but either genetic groups 2 or 3 based on the polymorphic regions of the *katG* and *gyrA* genes. Samples from three individuals in a family group were analysed further using PCR for *mtp-40*, *oxyR*, *plcD*, MT18101-*plcD*, as well as the RD7 deletion, in an attempt to distinguish *M. tuberculosis* from *M. bovis*. Molecular fingerprinting of the MTBC DNA was carried out using spoligotyping. The results of the *oxyR* pseudogene (guanine at position 185), *mtp-40* (positive), *plcD*-cutinase (positive) and RD7 (no product obtained) indicated the presence of *M. tuberculosis* rather than *M. bovis* DNA. Moreover, spoligotyping patterns obtained from all samples demonstrated the presence of *M. tuberculosis* DNA: one sample matched *M. tuberculosis* type 53 and two samples matched the profile of *M. tuberculosis* type 50 [65]. One of the individuals in this family group gave excellent results in a recent study based on shotgun sequencing and WGS [34]. A follow-up study based on 26 bodies from the Vác crypt demonstrated that all were of lineage 4, associated with European populations. Eight individuals were examined in depth and five had mixed *M. tuberculosis* infections with two, or in one case, three strains of different sub-lineages [66].

In 2003, Mays and Taylor described a case of tuberculosis from prehistoric times, which may be the oldest from Britain. They investigated remains excavated at the archeological site in Tarrant Hinton, England dated to 400–230 BC, which belonged to an individual suffering from an infectious disease, most likely tuberculosis. Nested PCR of the multi-copy element IS*6110* was performed in an attempt to confirm the presence of MTBC DNA and, hence, the diagnosis of tuberculosis. Four separate DNA extracts were prepared from bone powder sampled from vertebrae and ribs, and three of them were positive. Furthermore, two extracts of DNA from burials without any evidence of infection, which were studied as control samples, yielded negative results. PCR analysis for *Brucella* DNA was also undertaken using the IS*6501* multi-copy insertion element. Samples positive for MTBC DNA were further examined for polymorphisms using the *oxyR* pseudogene and the RD7 deletion, to distinguish *M. bovis*, *M. africanum* and *M. microti* from *M. tuberculosis*. In an attempt to provide information about the genotypic group, extracts were analysed for polymorphic sites in the *katG* 463, *katG* 203 and *gyrA* loci. Only one marker, IS*6110*, was efficiently amplified, probably due to the poor degree of DNA preservation [67]. This same case was re-examined in 2005 [39] in order to determine whether the Tarrant Hinton individual had suffered from tuberculosis due to *M. tuberculosis* or *M. bovis* and to perform further genotypic analysis of the Iron Age strain. To confirm that extracts prepared for this study were also positive for MTBC DNA, nested PCR for IS*6110* was carried out. Amplification of the IS*1081* sequence yielded positive results. Furthermore, two loci frequently used for differentiation between *M. tuberculosis* and *M. bovis* strains (*oxyR* and *pncA*) were analysed and gave positive results. Sequencing of the obtained PCR products showed guanine at position 285 (*oxyR* locus) and adenine at position 169 (*pncA*). Both results confirmed that *M. tuberculosis* DNA rather than *M. bovis* DNA was present in the analysed sample. The *M. tuberculosis* D1 deletion locus was also examined and identified the strain as a ‘modern’ TbD1-deleted *M. tuberculosis* isolate [39].

Ten samples from 18th century burials in Kaiserebersdorf Castle in Austria, now in the collection at the Natural History Museum Department of Anthropology in Vienna, Austria were investigated for the presence of MTBC DNA. Three bone samples from the anatomical ‘Weisbach collection’ were used as positive controls and two bone samples from a site at Hainburg were used as negative controls. PCR amplifications targeting IS*6110*, IS*1081*, *oxyR*, *pncA* and TbD1 regions of MTBC DNA, followed by cloning and DNA sequencing were performed. Despite the fact that PCR products of appropriate size were obtained for several bone samples, only PCR targeting the IS*6110* region in one sample yielded a product with a sequence identical to the reference IS*6110* insertion sequence of *M. tuberculosis*. Other PCR products were likely the results of non-specific PCR amplification. This failure to obtain a positive diagnosis of *M. tuberculosis* DNA in human remains was probably due to the poor aDNA preservation status of these bones from museum collections [68]. The preservation of the aDNA in a sample is not related to its age but to the local environmental conditions over time. For example, IS*6110*, together with the repetitive element IS*1081*, was successfully used in the molecular analysis of Neolithic skeletons (5400–4800 BC) from central Germany [14] and Hungary [6], dating from 4970–4490 BC.

Bouwman et al. used an NGS approach which involved hybridisation capture directed at specific polymorphic fragments of the *M. tuberculosis* genome, as well as a conventional PCR approach, to genotype a historic strain of *M. tuberculosis* from an individual dated to the 19th century AD, originating from St. George’s Crypt in Leeds, West Yorkshire, England. The SNP data obtained enabled a comparison between this strain and extant isolates of *M. tuberculosis*. This enabled the assignment of the historic strain into lineage II, SCG 6b and SNP type (ST) 14 or 40. Thirteen loci successfully typed by the conventional PCR approach confirmed the accuracy of the NGS results, demonstrating that NGS is a valuable method that can be used in investigating ancient varieties of *M. tuberculosis* [31].

In 2014, Müller et al. carried out a biomolecular analysis of MTBC DNA isolated from the remains of individuals living between the 1st and 19th centuries AD found at 29 British sites (59 samples) and eight continental European sites (11 samples). Both IS*1081* quantitative PCR and IS*6110* PCR were performed on each sample. Twelve samples definitely contained MTBC DNA (IS*6110* PCR was positive in at least two extracts). However, the diagnosis of a further 22 cases was described as probable or possibly containing DNA of the MTBC (IS*6110* PCR or IS*1081* real-time PCR positive in one of the extracts). Forty-three other samples produced no amplicons from any of the targeted sequences [69]. Thirty-four bone and dental samples from 31 individuals with positive results of IS*6110* and IS*1081* PCRs were characterised further. Conventional PCR of 11 SNPs and two LSPs was performed in an attempt to classify MTBC strains into 1–3 principal genetic groups (PGGs; *gyrA* 284, *katG* 1388), I–IV lineages and *M. bovis* (*oxyR* 37, *oxyR* 285, *rpoB* 2646, *rpoB* 3243), 1–7 SCGs (*leuB*, *qcrB*, *recN*, Rv0083, Rv2802c), ‘modern’ *M. tuberculosis* (TbD1-deleted) and the Euro-American lineage of modern *M. tuberculosis* (*pks15/1*-deleted). Data obtained from ten samples allowed categorisation of the MTBC according to their SNP identities, appearance of the TbD1 locus and/or the occurrence of deletion within *pks15/1*. PCGs and SCGs were successfully defined in two independent extracts from six samples. The results are summarised in Table 5 [70].

**Table 5:** Results of ancient *M. tuberculosis* genotyping obtained in Manchester and Madrid from two independent extracts [70]

	TbD1	*pks15/1*	PGG	Lineage	SGG
Sample					
Ashburch 705	−/−	+/+	2 or 3/2	I or II/nd	3/3 or 4
Auldhame 43	−/×	+/+	2/2	II/nd	5/5
Saint Amé 20	nd/×	nd/+	3/3	Not IV/not III	6/6
St. George’s Crypt 4006	−/−	+/+	3/3	II/II	6/6
St. George’s Crypt 5003	nd/−	+/nd	nd/nd	Not IV/nd	6/nd
St. Peter’s Church 1390	−/×	+/+	2/2	I or II/nd	3/3
St. Peter’s Collegiate Church 28	−/×	+/nd	3/nd	I or II/nd	6/nd
St. Peter’s Collegiate Church 62	−/−	+/+	2/2	I or II/II	4/4
Whitefriars 657	nd/×	+/nd	2/3	I or II/nd	6/6
Whitefriars 10466	−/×	nd/+	2/2	I or II/nd	4/4

− = deletion of TbD1 has occurred; + = 7-bp deletion in *pks15/1* has occurred; nd = not determined; × = not analysed

Although recent studies have investigated whether human cases of tuberculosis have been caused by *M. bovis*, there is only one good example from Iron-Age south Siberia, where semi-nomadic pastoralists overwintered with their animals [71]. This was diagnosed by a genotypic analysis of the aDNA using PCR based on regions of difference (RD) sites. In another and unexpected finding, the use of NGS has revealed that 1000-year-old pre-Columbian Peruvian human skeletons were infected with a member of the MTBC that appears to be *M. pinnipedii*, which is normally associated with sea mammals. This raises many questions but it is suggested that these individuals, who lived by a river that flows to the Pacific Ocean, may have become infected by contact with seals [72].

In summary, it is clear that the detection and characterisation of aDNA from *M. tuberculosis*, *M. bovis* and the MTBC is well advanced, in line with the strong interest in the modern disease.

**Fig. 3:**
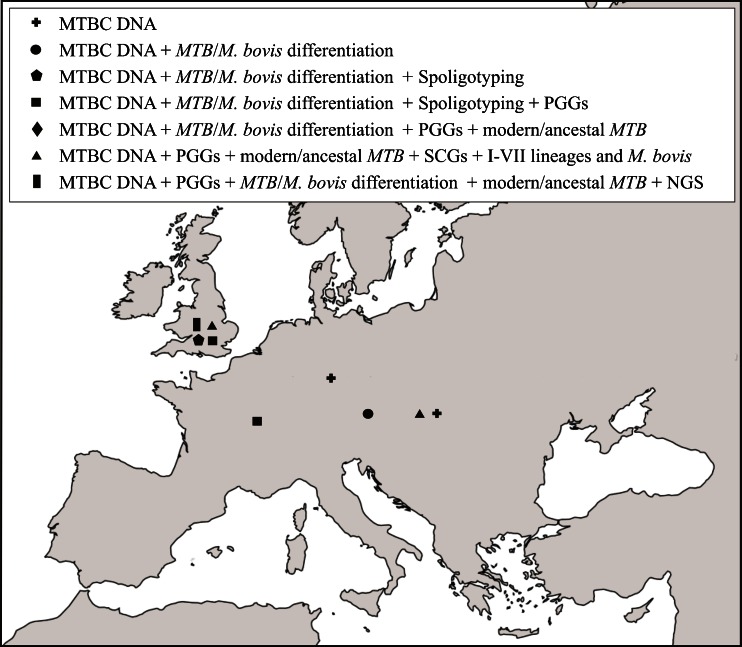
Examples of analytical methods used to identify MTBC aDNA in selected European sites

Figure 3 shows molecularly investigated ancient MTBC cases presented in this review.

### Molecular investigations of ancient leprosy

The first case of ancient leprosy to be subjected to molecular examination, dating from the first millennium, was obtained from a leprosarium, now in modern Israel [23, 24]. the results were initially queried, as the PCR target region was for a 530-bp target in the 36 kDa antigen locus. It was thought to be too large, but recent studies illustrate the remarkable preservation of *M. leprae* aDNA in some samples [33]. Another individual case of leprosy investigated by this group used PCR as paleopathologists could not agree on the cause of the visible bony lesions [73]. Meanwhile, Haas et al. from Münich undertook a molecular study of leprosy from a 10th century cemetery in Hungary and a South German ossuary of a later date [74], basing their samples on bone paleopathology. *M. leprae* DNA was amplified and sequenced. In addition, evidence was obtained of *M. tuberculosis* in another specimen.

The first recognised case of leprosy in Mediaeval Poland, from Suraż, dated to the 12th–14th centuries AD [4]. This case was included in a molecular study alongside archaeological samples with lesions from Hungary (one sample dated to 14th–15th century AD and two samples dated to 10th–11th century AD). *M. leprae* nested PCR for the specific repetitive element RLEP (36 copies per cell) and the 18 kDa antigen gene (single copy per cell), followed by DNA sequencing, were used to detect the target aDNA. This was successfully achieved using extracts from cavum nasale samples [75].

The first British case of leprosy to be subjected to molecular analysis, for the RLEP region, was from a mid-10th century burial in a Norse Christian cemetery in Orkney, Scotland [76]. In 2006, Taylor et al. examined two individuals, with demonstrable osteological evidence of lepromatous leprosy, from two English Mediaeval sites: Wharram Percy, Yorkshire (burial G708 radiocarbon dated to 960–1100 AD) and Blackfriars Friary, at Ipswich, Suffolk (burial 1914 radiocarbon dated to 1263–1538 AD). Again, the sensitive multicopy RLEP PCR was used to confirm the presence of *M. leprae* DNA and both burials were found to be positive. VNTR typing was used to assess genetic variability between strains in these cases and also a modern reference strain of *M. leprae*. The microsatellite regions, AGT and TTC, and one minisatellite region containing exact copies of a 21-bp repeat, were genotyped. The VNTR analysis (Table 6) and genotyping demonstrates differences between modern and historical *M. leprae* and between the two strains from investigated burials [77].

**Table 6:** VNTR typing of two medieval leprosy cases from Wharram Percy and Blackfriars Friary compared to modern leprosy DNA [77]

	Individual from Wharram Percy	Individual from Blackfriars Friary	Modern DNA
	Number of repeats		
AGT	8	9	10
TTC	10	12	13
21-bp repeat	3	2	2

Watson et al. sought evidence of *M. leprae* DNA in ancient European remains from Croatia, Denmark and the UK. They were dated to 476–1350 AD and examined by RLEP PCR and SNP typing. RLEP PCR was positive in three samples from the UK, one sample from Denmark and two samples from Croatia. DNA extracts that were positive for the RLEP sequence were further typed. All examined samples were of SNP type 3 [78] (corrected 2010 [79]). The genotyping study of both modern and ancient strains of *M. leprae* [55] identified the Hungarian strains as genotype 3K or 3M. In 2011, Taylor and Donoghue used three variable VNTR loci, AGT, TTC and 21–3, to characterise *M. leprae* from five archeological sites with a confirmed presence of *M. leprae* DNA and known SNP types and subtypes: Hungary, cases 503 and 222 (10th century), KD271 (7th century); the Czech Republic, burial 188 (9th–10th century) and northern Byzantine Turkey, KK ’02 20/1 (8th–9th century). All the examined samples were found to have distinct MLVA profiles. Furthermore, an individual (burial 1914, 13th–16th century) from Ipswich, Suffolk [77] was genotyped as SNP subtype 3I [80]. The 2006 study [77] also confirmed that a leprosy case from Uzbekistan, dated to the 1st–4th centuries AD, was of genotype 3 L, the only known example from aDNA analysis.

Rubini et al. examined two cases of childhood leprosy from the ancient Roman and Byzantine Empire. The first case was a skeleton of a 4–5-year-old child from a Roman cemetery at Martellona dated to the 2nd–3rd centuries AD. The second case, a 4–5-month-old infant was from a burial at Kovuklukaya in northern Byzantine Turkey, as mentioned above. Both skeletons exhibited bony changes indicative of leprosy. RLEP PCR was positive only for the second case and, at present, it is the youngest individual in the world proved to have suffered from leprosy in the past [81].

An early molecular study of leprosy in Sweden [82] demonstrated *M. leprae* aDNA from the Viking era. Subsequently, Economou et al. examined ten skeletons, from two cemeteries in Sigtuna, Sweden dated to the 9th–13th century AD, for *M. leprae* genomic sequences. Eight of those individuals had formerly been described as exhibiting bone changes characteristic for leprosy. RLEP PCR analysis was performed and nine specimens were positive for the repetitive RLEP element. SNP typing was possible in three samples: one was characterised as SNP type 3I and two as SNP type 2F. The remaining specimens either yielded no amplicons or results for one or two loci only. SNP type 3I has been described in a number of cases from ancient Europe. SNP type 2F had never been reported in Europe before and was previously considered as Asian [83]. Taylor et al. sampled nine skeletons showing good osteological signs of leprosy and bone preservation, originating from the St. Mary Magdalene leprosy hospital site in Winchester, UK. Two skeletons with no signs of leprosy were also sampled as negative controls. The presence of *M. leprae* DNA was investigated using the multicopy element RLEP, as well as the single copy 18 kDa antigen gene. Samples were subsequently characterised by SNP and MLVA typing. The results of *M. leprae* fingerprinting are shown in Table 7. Two SNP types were obtained during studies: 3I-1 and 2F. SNP type 3I-1 has been observed previously in the UK and confirms the association of SNP type 3 strains with Europe. However, SNP type 2F has never been described in the UK before and has formerly been reported in Asia and lately in Scandinavia [84]. Other cases from England and Scandinavia also appear to be predominantly of genotypes 2F or 3I [33, 85], suggesting that SNP types 2 and 3 co-existed in Mediaeval Europe.

**Table 7:** Genotyping of nine individuals from the St. Mary Magdalene leprosy hospital site in Winchester, UK [84]

	RLEP	18 kDa antigen	SNP typing	MLVA typing			
				AGA(20)	(GTA)9	21-3 loci	IS1081*
Negative control							
Sk1	−	−	×	×	×	×	−
Sk12	−	−	×	×	×	×	−
Samples							
Sk2	+	+	3I-1	11	8	2	−
Sk7	+	+*	3I-1	13	8	2	−
Sk8	+	+	2F	14	8	2	−
Sk9	+*	−	×	×	×	×	−
Sk14	+	+	2F	14	8	2	−
Sk15	+*	−	×	×	×	×	−
Sk18	+*	−	×	×	×	×	−
Sk19	+	+*	3I-1	14	7	Fail	−
Sk23	+*	+*	×	×	×	×	−

− = no result; + = positive result; +* = weakly positive result; × = no analysis was done

DNA bead capture and high-throughput sequencing (NGS) were used by Schuenemann et al. to obtain complete genome sequences of *M. leprae* from skeletons from five Mediaeval archeological sites in the UK, Sweden and Denmark. These were compared with 11 modern strains. Bayesian phylogeny inference, maximum likelihood, neighbour joining and maximum parsimony were used to calculate the phylogenetic trees of all genomes. The majority of the examined strains clustered into four major branches supported by bootstrap values of >90 % for all major nodes. This is consistent with the SNP typing scheme. Two modern strains had the deepest lineage and formed a new branch 0. One modern strain displayed deep divergence and did not fit definitely into one branch. These three different strains were found to be those closest to the suggested common ancestor of *M. tuberculosis*, *M. leprae*, *M. avium* and *M. ulcerans*. The nucleotide distance of 16 sequences to the reconstructed most recent common ancestor (MRCA) of all *M. leprae* strains was calculated in an attempt to verify if the ancient *M. leprae* strains have shorter branches in a phylogenetic tree compared to the modern branches. The average distance to the MRCA for the ancient strains was 19.8 nucleotides and for the modern strains it was 27.5 nucleotides. The ancient strains displayed significantly shorter branches in the tree. Strains assigned to branch 2 formed a tight cluster with very short branch lengths. Branch 3 strains are found in European ancient samples and in modern samples from America, indicating the European origin of leprosy in the Americas. SNP subtyping was performed also for individuals from 11th–14th century AD, Denmark and assigned to 3I and 2F groups, the same as that reported by others [33, 83, 84]. Variations in the *M. leprae* subtype 3I have been found [84] and, in a recent study of a 5th–6th century male from Essex, East England [86], the authors suggest that this *M. leprae* strain was ancestral to the Mediaeval strains and may have originated in Scandinavia. Using collated evidence of the distribution of *M. leprae* in ancient and historical Europe, based on genotyping where possible, Donoghue et al. have suggested that the differences between subtypes in central and east Europe, compared with northwest Europe, may be linked to human migration [5].

**Fig. 4:**
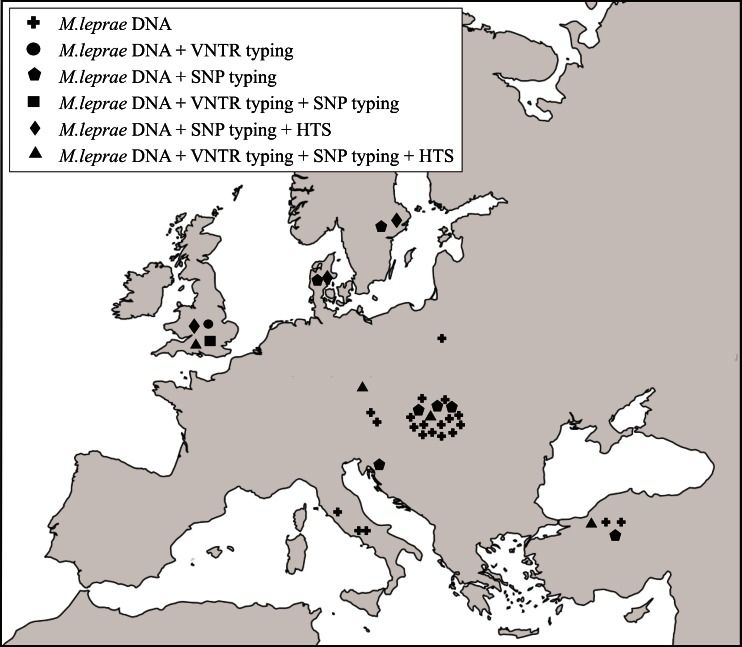
Examples of analytical methods used to identify *M. leprae* aDNA in selected European sites

All molecularly investigated cases of ancient leprosy described in the review are presented in Fig. 4.

## Significance of the host genome in mycobacterial diseases

Investigation of the aDNA from human pathogens enables the assessment of disease epidemiology in past millennia. Development of mycobacterial diseases, as for many other infectious conditions, depends on both environmental and genetic factors, i.e. host and pathogen genomes. Only about 10 % of carriers of *M. tuberculosis* develop the disease, thereby indicating the importance of alleles coding for resistance in the formation of a pathogen-resistant phenotype [87]. However, it should also be remembered that other non-genetic factors influence human susceptibility to infection, such as co-morbidity, co-infections, dietary deficiencies, stress and trauma [88]. The significance of the host genome in the development of mycobacterial diseases has been confirmed by studies on mono- and di-zygotic twins and by documented differences in disease incidence around the world, correlated with the history of the disease in different regions [89]. The immunological resistance to leprosy and tuberculosis has developed under strong selective pressures, such as epidemics, which have promoted the survival of the best-adapted individuals and changed the composition of the gene pool, i.e. increased the frequency of resistance-related alleles. Therefore, the presence of pathogens in human populations has left a strong mark and the differences between ancient and present gene pools can elucidate the course of major epidemics and help resolve any doubts about the time of disease origin. Moreover, studies based on changes in the frequency of resistance-related alleles provide information about whole populations rather than individuals, where data are gained by the examination of pathological bone lesions. A broader paleopathological approach that combines population genetics with the molecular analysis of pathogens responsible for infectious disease constitutes a promising tool of much greater power in reconstructing human history rather than using a single-approach analysis.

### Genes involved in genetic predisposition to leprosy and tuberculosis

Genetic predisposition to tuberculosis and leprosy is still a subject of extensive investigation due to the complexity of reactions induced by mycobacteria. Owing to intensive research including family-based and case–control studies, several polymorphisms can be linked to mycobacterial diseases. Candidate genes linked to a tuberculosis-susceptible genotype involve a great number of immunological response components, such as cytokines, chemokines and receptors. However, some genes with influence on tuberculosis development are well investigated and confirmed. Examples are genetic alleles of mannose-binding lectin (*MBL2*), interleukins (*IL1*, *IL6*, *IL8*, *IL10*), interferon (*IFNG*), receptors (*PR2X7*, *TLR1*, *TLR2*) and human leukocyte antigens [87, 90, 91]. Nevertheless, the strongest evidence for genetic susceptibility to both tuberculosis and leprosy is based on the solute carrier family 11 *SLC11A1*, formerly known as natural resistance-associated macrophage protein 1 (*NRMAP1*) [92]. It encodes a divalent cation transporter build of 550 amino acids which is recruited to the phagolysosomal membrane during phagocytosis in macrophages and neutrophils [93].

Killer cell immunoglobulin-like receptors (KIRs) are members of a group of regulatory molecules found on subsets of lymphoid cells, first identified by their ability to impart some specificity on natural killer (NK) cytolysis. The KIR locus, which maps to chromosome 19q(13.4) within the 1 Mb leukocyte receptor complex (LRC), contains a family of polymorphic and highly homologous genes. The KIR molecules recognise the human leukocyte antigen (HLA) class I molecules, which are encoded by genes within the major histocompatibility complex (MHC) chromosome 6 [94]. Interactions occur between KIR isotypes that inhibit NK cell activity. Recent studies report a greater repertoire of inhibitory KIR genes among tuberculosis patients than controls [95] and a direct association of certain KIR and HLA-C genes [96] with resistance to pulmonary tuberculosis.

Several *SLC11A1* polymorphisms (e.g. 3′UTR, D543N, 5′ (GT)n, INT4) have been linked to host genetic predisposition to both leprosy and tuberculosis in various populations, making it a common rather than a local susceptibility-related gene. To estimate the role of this ion transporter in forming resistant genotypes, Barnes et al. [97] determined the frequency of one allele (SLC11A1 1729 + 55del4) in various populations from global cities with a different length of time since urbanisation. Interestingly, in longer-term urbanised populations, the incidence of the protective allele was significantly higher as a result of natural selection. Their studies support the thesis that urbanisation and population density determine the general health status of the population and influence its genetic structure. Moreover, an initial study on the comparison of allele frequencies (*SLC11A1*, *MBL2*) that formed part of a Ph.D. dissertation revealed interesting discrepancies between medieval and contemporary Polish populations [98]. Samples from various 11th–14th century archaeological sites displayed a consistent pattern of a significantly higher frequency of alleles predisposing to infectious diseases compared with the contemporary Polish population. The results point to a positive selection of protective variants over time, most probably due to past epidemics.

Susceptibility to leprosy depends on various mutations within several genes, for example, the major histocompatibility complex (*HLA-DR*, *HLA-DQ*), tumour necrosis factor α (*TNFA*), Toll-like receptors (*TLR1*, *TLR2*, *TLR4*, *TLR9*), vitamin D receptor (*VDR*) or cytotoxic T cell antigen 4 (*CTLA4*), among others [89, 99, 100]. However, the predisposition to mycobacterial diseases depends upon the ethnic group, which makes it difficult to determine one genotype responsible for susceptibility to leprosy. The best-described leprosy-related genetic markers are regulatory polymorphisms within *PARK2*/*PACRG* genes. Even though the function of the *Parkin* gene is still unknown, it has been proved that two polymorphisms within the 6q25 locus are involved in susceptibility to paucibacillary and multibacillary leprosy [101, 102]. Bakija-Konsuo et al. measured the frequency of selected changes in the isolated population of Mljet island in Croatia, used in the past to quarantine for leprosy patients, and compared it with the reference populations with no history of leprosy [103]. They found a significant increase in the frequency of both protective alleles, indicating their positive selection in the course of high disease prevalence and demonstrating the importance of host genotype and changes in population gene pools under high selective pressure.

According to John Haldane, 1949, cited by Bellamy in 2005 [90], pathogens have been the major agents for natural selection over the last 5000 years. He suggested that natural selection was driven mainly by the necessity to resist pathogens, as they were the main cause of premature deaths in the past [90]. Between the 18th and 19th centuries, during the Industrial Revolution in Europe, fast development and the human population explosion, as well as the probable increase of pathogen virulence, initiated a huge increase in tuberculosis, which caused one-fifth of all deaths. The low incidence of tuberculosis in Europe in the recent past indicates that the development of tuberculosis depends predominantly on the host genome rather than the pathogen. Therefore, the frequencies of selected alleles that predispose to tuberculosis and leprosy among past populations of different ages could elucidate the susceptibility/resistance status of selected populations and, thus, the presence of pathogenic mycobacteria in that period. Even though the history of both tuberculosis and leprosy is moderately understood, there are still many aspects that require resolution, including the time of the emergence of these mycobacterial diseases in Europe. The comparison of various populations can provide data about the dynamics of change and distinguish the role of each allele in the process of resistant phenotype formation. Thus, further research on host genes related to susceptibility/resistance to mycobacterial diseases is crucial in order to identify the mycobacterial resistant genotype in different populations and enabling the recognition of changes in allele frequencies. Extending this to aDNA studies, limited data are now available for amplified aDNA from 18 individuals from the 18th century Vác, Hungary and early Christian Nubia [88].

### Other applications of susceptibility studies

Research on genetic susceptibility among ancient populations complements work on paleoepidemiology. These have mainly focused on the pathogen, and have already been successfully employed in several studies. In 2006, Witas et al. assessed the frequency of the *Δ32-CCR5* mutation in a medieval Polish population and compared it with the contemporary gene pool [104, 105]. The deletion of a 32-bp sequence in the coding region of this chemokine receptor leads to the perturbation of its fusion to the cell membrane [106]. The lack of two functional copies of the *CCR5* gene results in the absence of the leucocyte surface receptor and results in a strong but incomplete resistance to HIV-1 infection. The area of mutation occurrence is restricted mainly to Europe and parts of Asia. Its frequency in Europe varies according to the region, from 4 % in Sardinia to 15 % in Finland [107]. In present-day Poland, the incidence is similar to the European average of 10.9 % [108]. However, the frequency of this protective allele in the medieval Polish population (11th–14th century) was half as great (5.06 %), which may signal the positive selection due to the protecting allele during this time period [104, 105]. These results conflict with the alternative suggestion that *Yersinia pestis* was a selective factor for *Δ32-CCR5*, as the timing of plague epidemics does not match the likely emergence of the deletion and the origin of this mutation remains unsolved. Meanwhile, Fontecchio et al. have attempted to answer the question about the susceptibility to rheumatoid arthritis (RA) in the decay of the Middle Ages. It is commonly believed that the disease was brought to Europe from America at the end of the 15th century. The genetic predisposition to RA, based on the polymorphic HLA-DRB1 locus, which was carried by an early case known as the ‘Braids Lady’ from Arezzo (Italy), combined with pathomorphological lesions characteristic for this condition. This confirms the presence of RA in the Old World in the mid-16th century and brings us closer to answering the question about the worldwide diffusion of rheumatoid arthritis [109].

## Summary

Emerging molecular approaches employed in the investigation of *M. tuberculosis* and *M. leprae* in ancient human remains began a new era of paleopathological analysis. The techniques described have been applied successfully in several European studies, revolutionised the classical approach to ancient disease studies and accelerated the development of paleomicrobiology. As the molecular analysis of bacteria from ancient material has become widespread, it is possible to determine their phylogenetic affiliation. Therefore, studies of bacteria from different times and locations will undoubtedly enable a better understanding of the origin of infectious diseases. The novel techniques of aDNA sequencing have enabled the identification of genes related to susceptibility and resistance phenotypes within ancient populations, which may be an important factor in the determination of past bacterial prevalence. Methods have been reviewed and changes in population immunological profiles over time are assessed. The overall aim is to reach a better understanding of the history of tuberculosis and leprosy and to determine the timescale of major outbreaks. However, the complexity of the immune response induced by mycobacterial infections, such as the great number of gene polymorphisms involved in host susceptibility, constitutes a major problem in employing such methods in paleoepidemiology. Nevertheless, the progress in elucidating host genetic profiles, linked to the predisposition of susceptibility or resistance to infection, is enabling a better understanding of the course of two major historical diseases in the human population.
